# The Imaging Probe Development Center and the Production of Molecular Imaging Probes

**DOI:** 10.2174/1875397300801010065

**Published:** 2008-06-13

**Authors:** Gary L Griffiths

**Affiliations:** Director, Imaging Probe Development Center, National Heart, Lung, and Blood Institute, NIH, Bethesda, MD, USA

## Abstract

The Imaging Probe Development Center (IPDC), part of the NIH Roadmap for Medical Research Initiative (http://nihroadmap.nih.gov/) recently became fully operational at its newly refurbished laboratories in Rockville, MD. The IPDC (http://nihroadmap.nih.gov/molecularlibraries/ipdc/) is dedicated to the production of known and novel molecular imaging probes, with its services currently being used by the NIH intramural community, although in the future it is intended that the extramural community will also benefit from the IPDC’s resources. The Center has been set up with the belief that molecular imaging, and the probe chemistry that underpins it, will constitute key technologies going forward. As part of the larger molecular libraries and imaging initiative, it is planned that the IPDC will work closely with scientists from the molecular libraries effort. Probes produced at the IPDC include optical, radionuclide and magnetic resonance agents and may encompass any type of contrast agent. As IPDC is a trans-NIH resource it can serve each of the 27 Institutes and Centers that comprise NIH so its influence can be expected to impact widely different subjects and disease conditions spanning biological research. IPDC is expected to play a key part in interdisciplinary collaborative imaging projects and to support translational R&D from basic research through clinical development, for all of the imaging modalities. Examples of probes already prepared or under preparation are outlined to illustrate the breadth of the chemistries undertaken together with a reference outline of the diverse biological applications for which the various probes are intended.

## BACKGROUND

In late 2004 the US National Institutes of Health launched its Roadmap for Medical Research in the 21^st^ Century (http://nihroadmap.nih.gov/). One of three major themes was identified as “New Pathways to Discovery” one of which, in turn, comprised the Molecular Libraries and Imaging Initiative (http://nihroadmap.nih.gov/molecularlibraries/). The Libraries Roadmap Initiatives have put in place personnel and facilities which enable the large-scale screening of hundreds of thousands of small molecules as molecular probes which can be used to study the full spectrum of cellular and biochemical processes [1]. Data obtained from this massive effort is made available to the public and to biomedical researchers throughout the world, and this unprecedented access to shared information is expected to greatly facilitate new drug development efforts, even for heretofore overlooked and/or rare diseases [2,3]. This approach has been enabled by three disparate complimentary technologies reaching maturity, namely the elucidation of the complete human genome, advanced robotics and informatics, and high throughput chemistry and assay development, leading to the emergence of ‘chemical genomics as a vital new field of study’ [4].

Concomitant with, and complementary to, the Libraries Initiatives are the Roadmap Molecular Imaging Initiatives which include an imaging agent database (MICAD), an extramural research effort aimed to dramatically increase the sensitivity and specificity of molecular imaging agents, and a core chemistry facility to actually synthesize imaging probes – the Imaging Probe Development Center (IPDC) (http:// nihroadmap.nih.gov/molecularlibraries/ipdc/). The field of molecular imaging has also been undergoing profound changes in the last few years spurred by advances in detection capabilities in the major modalities of magnetic resonance imaging, radionuclide imaging using both single photon and positron emitting nuclides, and most notably, in fluorescence microscopy [5-12]. One aspect crucial to the continuing development, broader use and applied clinical translation of these modalities is the availability of molecular probes, which often need to be synthesized in a tailored fashion for each particular application. Recognition of the basic underlying need for access to applied chemistry for the production of diverse imaging probes was acknowledged as a significant barrier to progress in molecular imaging in the initial Roadmap discussions and led directly to the formation of the IPDC. In a ‘needs’ analysis prior to IPDC formation it was noted that only a few small to intermediate size companies (e.g. Invitrogen, Macrocyclics, etc.) provided specialized agents for molecular imaging studies and even then specialized requests such as a one-time synthesis or modest synthetic runs for testing of even known imaging probes were very difficult to secure. Although some academic laboratories have capacity to generate probes for their own studies they are very limited in any capacity to provide them for other laboratories, leading to a bottleneck in the continuation of promising studies by other academic researchers. Finally, there was no existing NIH program designed to provide a wide range of optical, PET, SPECT, or MRI imaging probes to academic investigators.

The goal of the IPDC, therefore, is to provide a mechanism to produce “known” or published agents for which there is no commercial supply, and to generate novel imaging probes, for biomedical research and clinical applications. The IPDC is composed of two major elements, (a) receptor modeling/biophysical chemistry, and (b) organic synthesis and it is intended that IPDC will complement and support the screening centers set up under the Libraries initiative since molecular imaging can be seen as a logical downstream application of the screening centers discovery efforts. We began operations after moving into a newly refurbished facility near the end of 2006, and initially solicited proposals for new imaging probe compositions from the NIH intramural community. At the same time we recruited a staff of young scientists with diverse interests and expertise related to imaging agents and centered on one or more of classical synthetic, polymer, bioconjugate, metal complexation chemistries or radiochemistry. Along with the new staff, multiple new pieces of equipment {Flash, HPLC, FPLC, MS – ES,-CI, -MALDI, NMRs, etc.} were obtained, installed and trained on for the simultaneous preparation of any type of composition we might be asked to produce in the coming years. In addition to the chemistry production, IPDC has biological evaluation facilities which will enable it to perform preliminary *in vitro* testing work, for instance, with binding of agents to cellular and protein receptors and substrate turnover studies in enzyme activity assays. A range of equipment for cell culture, biosafety cabinets and a variety of radiographic and fluorescence imagers including confocal microscopy support this biological aspect of the IPDC. This ‘overlap’ with end-user capabilities is often of great practical significance as a checking mechanism in inter-disciplinary work performed in different remote laboratories, smoothing progress by rapidly overcoming disputable inconsistencies in data, which may be seen from different laboratories merely by their use of different methods and different equipment. On the molecular modeling front IPDC scientists have access to the Helix, Biowulf and Double Helix super-cluster computers through the NIH Center for Information Technology. This enables scientists to study large molecule interactions and small molecule-protein docking studies through the AutoDock program. In addition molecular and quantum dynamics studies can be performed with smaller molecules to determine, for instance, how well sub-units of various molecules interact, as these properties are often important in overall fluorescent properties such as fluorescent lifetimes and fluorescent resonance energy transfer (FRET). It is clearly of vital importance that low molecular weight binding entities identified from screens and optimized for binding *via* analog syntheses are then only modified *via* chemical handles in positions that have minimal adverse effects on their target binding capabilities as well as on the detectable agent’s properties.

Agents used for molecular imaging at this point in time can run the full spectrum from low molecular weight drug-like molecules through natural products, peptides, nucleotides, sugars and their oligo- and combined conjugates, polymeric, proteinaceous and nanoparticle-like moieties, and more; all tagged with one or more of MRI, radionuclidic, ultrasound or fluorescently detectable labels.

## IPDC OPERATIONS

IPDC operates under the direction of a steering committee with members drawn from chemists and imaging specialists representing most of the Institutes with an interest in imaging and/or chemistry. The current make up of this committee in shown in Table **1** and represents a broad array of interests both with regard to human conditions/diseases and areas of basic science studies. All proposals are discussed in detail at monthly steering committee meetings prior to any acceptance or commitment on the part of the IPDC. Proposals submitted to the IPDC from the intramural NIH community must first be approved for submission by the Scientific Director of the Institute wherein the submitting scientist is located. In addition, IPDC is overseen by the Roadmap’s Office and Portfolio Analysis and Strategic Planning (OPASI), which oversees the entire Roadmap program, and monitors the IPDC through the Molecular Libraries and Imaging Implementation Group (MLIIG). The MLIIG, in turn and as the title implies, oversees the molecular libraries and imaging programs. Under these umbrellas IPDC sent out three solicitations for new project proposals from the NIH intramural community during its first year or so of existence.

## IPDC PROJECTS

Very strong interest and a very strong response throughout our first year led to the adoption of several dozen projects for the supply of imaging probes to multiple Institutes and Centers within NIH [13]. Over thirty of these projects are still ongoing at the time of writing and these will give rise to well over 70 individualized probe compositions. The projects undertaken by IPDC are broken down by requesting Institutes or Centers in Fig. (**1**), with the largest number of projects undertaken on behalf of the National Cancer Institute (NCI). Probe requests have covered the full spectra of chemical compositions used for molecular targeting from small molecular weight compounds from medicinal and natural products chemists through peptides, nucleotides, sugars and their analogs and oligomers, to compositions based on proteins, antibodies and their constructs, dendrimers, nanoparticles and liposomes. The detectable agent most requested for compositions is based on fluorescence as an imaging beacon, although a significant fraction is related to metal complexes for MRI studies and radionuclide-based agents.

Table **2** lists a selection of the probes made or under preparation by composition, detectable agent, the major requested indication, and NIH Institute. Known fluorescent analogs such as biarsenicals used for site-specific tetracysteinyl-protein tagging, which are tricky to make even for a well-trained chemist, have now been requested by several groups so that around half-a- dozen analogs have been produced. Requested originally for prion protein infection studies, they clearly can be applied as a research tool for any indication using a tetracysteinyl-labeling strategy. Fluorescent dyes are generally quite difficult to work with on a chemical basis as such preparations are generally more impure than most chemical starting materials, with confounding mixtures often involving several similar compounds as well as a selection of isomeric variants, none of which is usually of importance to a biological stain- or assay-minded biologist. Many fluorescent dyes are therefore repurified routinely prior to attempting further chemical manipulations.

Also widely requested are targeting agents using common dyes such as the Alexa and cyanine series, for instance cholesterol-linked dyes for lipid raft studies and GABA analogs for calcium channel neurotransmitters. Others are involved in basic studies designed to improve dye properties such as by modifying fluorescent lifetimes. Particularly interesting probes seek to dramatically improve fluorescent imaging by applying ‘caged’ agents, that is, agents that are non-fluorescent until an appropriate light source renders them so. Such agents are designed to make a dramatic leap forward in fluorescent imaging technology by increasing sensitivity toward tracking single molecules using photoactivation localization microscopy (PALM). PALM may allow researchers to break the diffraction barrier limit by selectively activating and detecting one molecule at a time, repeating the procedure numerous times, and then reconstructing a whole image from the accrued data. A different approach seeks to make a similar leap toward ‘super-resolution’ by using a stimulated emission depletion-like (STED) strategy employing Förster resonance energy transfer (FRET) pairs. The activatable FRET donor-acceptor technique is also applied in several other studies, for instance to probe HIV-1 infection mechanisms. This may have general implications for all diseases involving similar viral replication pathways where it greatly improves the target-to-background ratio. A separate and important series of caged probes are non-fluorescent caged glutamates which are used in receptor activation studies.

Intracellular event studies, outside of infection, are also well represented among our probe requests with enzyme substrates, for example, with a phosphatidylinositol-specific phospholipase C (PI-PLC) already being made. These complex and non-fluorescent multi-phosphatidylinositols will become fluorescent upon PLC cleavage and will offer a unique way to look at this important class of enzymes. Separately, we are working on specifically designed species to target intracellular compartments such as lysosomes and such agents can have an impact on all lysosomal storage diseases. This project was undertaken with the NCGC (the intramural component of the molecular libraries initiative and with whom we share our facility), who successfully identified targeting moieties of interest, and it represents the first example of these two new Roadmap initiatives working together on a common problem along with the requesting Institute.

Some fluorescent molecules may never be more than highly specialized research tools due to their being unavailable merely by reason of the difficulties involved in their syntheses. However, such agents may also offer special insights into basic cellular processes. Such a series of probes is illustrated by the pteridine nucleic acid analogs which are fluorescent analogs of the guanosine and adenosine nucleosides. Although very difficult to make, with up to 15 or more synthetic steps involved, often on highly labile intermediates, their unique capacity to be incorporated directly into the oligonucleotide chains of DNA and RNA could offer unique perspectives on basic oligonucleotide studies. They may even have future roles to play in studying the ‘small’ and ‘micro’ RNAs which are of so much current interest.

With MRI reagents the IPDC has been asked to prepare low and high molecular weight gadolinium complexes, manganese complexes and nitroxide radicals. These are generally used for *in vivo* studies, although some of the fluorescent agents mentioned can also be applied to animal imaging. The nitroxides are redox sensitive probes and are envisaged as agents for detecting the effects of radiation therapy. They are being tested in the context of radiotherapy for glioma. This agent potentially has broad application in monitoring radiation therapy. The manganese complexes are intended as bone imaging agents, as their chelate structures have appended phosphate groups, while it is also an interesting challenge from a chemist’s point-of-view as to whether a truly stable manganese-chelate complex for *in vivo* use can be obtained. The more common gadolinium ion has been attached to series of dendrimers using stable chelates for several applications including vascular and lymphatic imaging and as a new set of agents which may be able to traverse blood-brain-tumor barriers. Low molecular weight gadolinium-labeled agents are being studied as neural tracers to map connections within the brain.

Radiolabeled species represent the majority of animal and particularly human-use imaging probes. The IPDC is working on low molecular weight radiolabeled thyroid hormones for PET imaging of hypothyroid conditions, radiolabeled adenosine analogs for imaging adenosine receptors, radiolabeled dopamine receptor antagonists for dopamine receptor imaging, and radiolabeled vasopressin analogs for CNS receptor imaging. Proteins are also under study with serum amyloid and ‘Frizzled’ proteins being radiolabeled while ongoing radiolabeling work continues with antibodies. One satisfying aspect of the IPDC’s scope is that imaging sciences, more than most, bridges biotechnology and chemistry, and even includes nanotechnology as we are now producing nanoparticle-derived agents and are likely to do more of such work in the future. On a more compositional note we have also prepared targeting liposomes for use in studies targeting colon cancer. A notable example of our bridging, interdisciplinary position is our ongoing work with Affibody^®^ molecules, which are small enough to be chemically synthesized on a peptide synthesizer and also large enough to be produced from biotechnological cell fermentations. These picomolar affinity constructs represent a new platform for disease targeting, can be tagged with detectable agents from any of the modalities, and are being developed for both therapy and imaging uses.

## THE FUTURE

Molecular imaging is becoming ever more important in a broad range of studies being done from the smallest intracellular level through intact organisms, including humans. In addition, molecular imaging is being adopted by drug makers and biopharmaceutical firms as a useful adjunct to development work, diagnoses, therapy decision-making, and disease monitoring. Indeed, one might contend that many drugs of the future will need to use some kind of imaging work to justify their application, and as molecular imaging becomes ever more exact and sensitive that position can only be reinforced. It is intended that the IPDC will have the capacity to assist in the drug development process and in the transfer of new agents into initial clinical trials. Our capacity to make almost any reasonable imaging probe composition should make IPDC a useful resource, especially for scientists who may have no current chemistry experience or available collaborating chemists and may be blocked from carrying out what may be otherwise important investigations. But, as has also been demonstrated with our initial projects, even medicinal chemists can make use of the IPDC to do jobs that may be too difficult in their own laboratory, even if from just a regulatory viewpoint, as for instance with radiolabeling. The plethora of new agents being described in the literature clearly leaves many bioscientists frustrated that studies cannot be repeated and expanded in new directions often due to lack of chemistry resources, and the IPDC will seek to address this issue to the best of its abilities.

The IPDC looks forward to addressing the needs of the broader imaging community. The initial experience bodes well for the ability of IPDC to provide unique imaging probes that will provide biologic insights into the mechanisms of a broad spectrum of disease. Meanwhile I encourage those of you who are interested to feel free to contact me if you have questions about the IPDC or its services, or to enquire informally about a prospective molecular imaging probe that you may be interested in. I can be reached by email at:  griffithsgl@mail.nih.gov.

## Figures and Tables

**Fig. (1):**
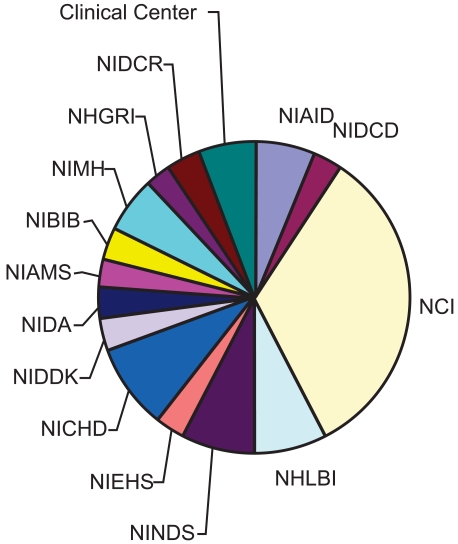
Distribution of IPDC projects between NIH intramural Institutes and Centers in February 2008. NCI shows the largest proportion with a total of 11 of the 34 ongoing projects.

**Table 1:** The Members of the IPDC Steering Committee in February 2008

Robert Balaban (NHLBI)	Allen Braun (NIDCD)
Martin Brechbiel (NCI)	Henry Bryant (CC)
Peter Choyke (NCI)	Julie Dyall (NIAID)
Amir Gandjbakhche (NICHD)	Gary Griffiths (NHLBI)
Daniel Hommer (NIAAA)	Robert Innis (NIMH)
Jerry Jennings (NIAID)	Yong Sok Lee (CIT)
Roderic Pettigrew (NIBIB)	Kenner Rice (NIDA)
James Sellers (NHLBI)	Richard Siegel (NIAMS)
Afonso Silva (NINDS)	Craig Thomas (NHGRI)

**Table 2:** A Selection of the Molecular Imaging Probes Made or Being Made, their Intended Application, and the NIH Institute Initializing the Request

Composition(s)	Refs	Detectable Agent(s)	Indication(s)	NIH Institute
Biarsenicals	[14]	Fluorescent protein	Prion infection studies	NIAID
Thyroid hormones	[15]	Radioiodine	Thyroid disorders	NIDCD
Affibody^®^ molecules	[16]	Multiple	Cancer	NCI
Enzyme substrate	[17]	Fluorescent	PI-PLC	NHLBI
Dendrimer complexes	[18]	Gadolinium	Vascular studies	NCI
Caged glutamate	[19]	Fluorescent	Receptor activation	NINDS
Cholesterol derivatives	[20]	Fluorescent	Lipid rafts	NIEHS
Chelate complexes	[21]	Fluorescent	Lifetime modulation	NICHD
Receptor binding agents	[22]	MRI	Breast cancer imaging	NCI
Nucleotide analogs	[23]	Fluorescent	DNA/RNA probes	NCI
Adenosine analogs	[24]	Radioiodine	Receptor agents	NIDDK
Nitroxides	[25]	MRI	Radiation damage	NCI
Caged molecules	[26]	Fluorescent	Intracellular trafficking	NICHD
Dopamine antagonists	[27]	Radiolabeled	Neurological disorders	NIDA
Liposomes	[28]	Multiple	Colon cancer	CC
Dendrimer complexes	[29]	MRI	Gliomas	NCI
Proteins	[30]	Radiolabeled	Amyloid diseases	NIAMS
Receptor binding agents	[31]	Fluorescent	Calcium channel agents	NIA
Gold nanoparticles	[32]	X-ray	HIV-1 binding sites	NCI
Chelate complexes	[33]	Gadolinium	Neural trafficking	NIMH
Nucleotides	[34]	Fluorescent	HIV-1 studies	NCI
Peptides	[35]	Radiolabeled	Receptor studies	NIMH
Receptor binding agents	[3]	Fluorescent	Lysosomal diseases	NHGRI
